# Supplementary material for: Altered visual cortex excitability in premenstrual dysphoric disorder: Evidence from magnetoencephalographic gamma oscillations and perceptual suppression
Source: PLoS One. 2022 Dec 30;17(12):e0279868. doi: 10.1371/journal.pone.0279868 (PMC9803314; doi:10.1371/journal.pone.0279868)
Supplement: S1 Fig — Violin plots for gamma response (GR) power (upper panel) and GR frequency (lower panel). Note that the group differences were not significant for all of these GR parameters (t-test, all p’s>0.12; uncorrected for multiple comparisons). (DOCX) [file pone.0279868.s001.docx]

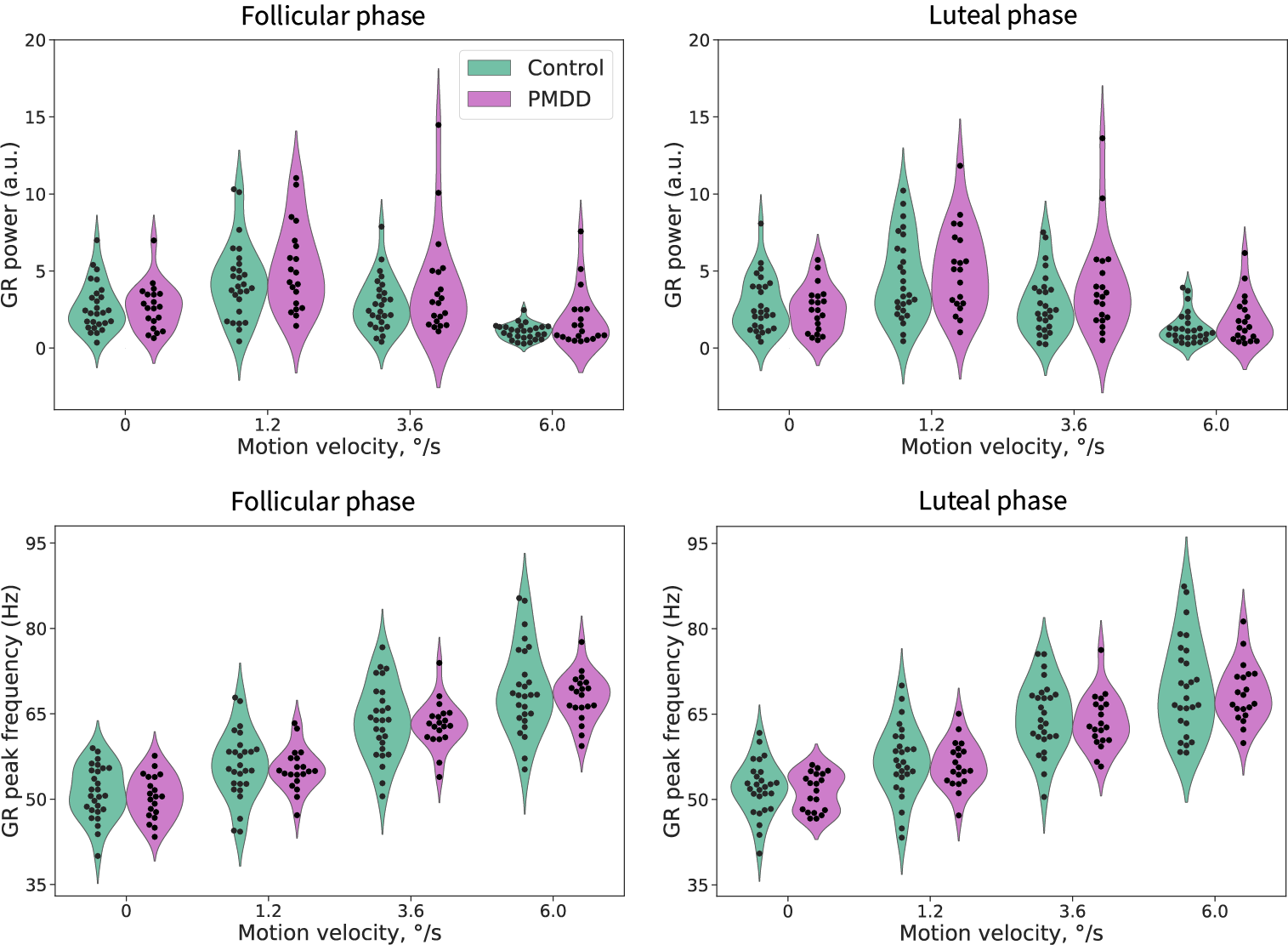
**S1 Fig. Violin plots for gamma response (GR) power (upper panel) and GR frequency (lower panel).** Note that the group differences were not significant for all of these GR parameters (t-test, all p’s>0.12; uncorrected for multiple comparisons).
