# Supplementary figures and images for: Altered visual cortex excitability in premenstrual dysphoric disorder: Evidence from magnetoencephalographic gamma oscillations and perceptual suppression
Source: PLoS One. 2022 Dec 30;17(12):e0279868. doi: 10.1371/journal.pone.0279868 (PMC9803314; doi:10.1371/journal.pone.0279868)

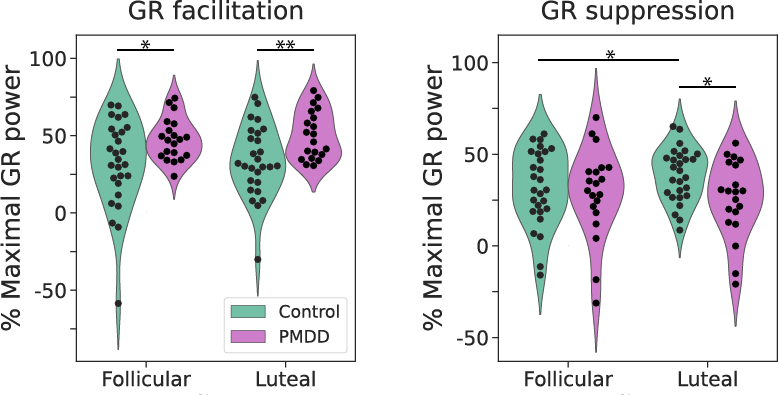


**S2 Fig. Violin plots of gamma response (GR) suppression and GR facilitation scores.**

Supplement: S2 Fig — (DOCX) [file pone.0279868.s002.docx]
