# Supplementary material for: Altered visual cortex excitability in premenstrual dysphoric disorder: Evidence from magnetoencephalographic gamma oscillations and perceptual suppression
Source: PLoS One. 2022 Dec 30;17(12):e0279868. doi: 10.1371/journal.pone.0279868 (PMC9803314; doi:10.1371/journal.pone.0279868)
Supplement: S1 File — (DOCX) [file pone.0279868.s003.docx]

**Supplementary Information**

**Altered visual cortex excitability in premenstrual dysphoric disorder: evidence from magnetoencephalographic gamma oscillations and perceptual suppression**

Viktoriya O. Manyukhina^1,2^, Elena V. Orekhova^1^*, Andrey O. Prokofyev ^1^, Tatiana S. Obukhova^1^, Tatiana A. Stroganova^1^

^1^ Center for Neurocognitive Research (MEG Center), Moscow State University of Psychology and Education, Moscow, Russian Federation.

^2^ National Research University Higher School of Economics, Moscow, Russian Federation.

* Corresponding author

Email: [orekhova.elena.v@gmail.com](mailto:orekhova.elena.v@gmail.com) (EO)

**A short running title:** Altered visual cortex excitability in PMDD

**Supplementary methods, materials and results**

**Results of bipolar disorder and depression questionnaires in women with PMDD**

The bipolar and depression questionnaire scores for the PMDD group are summarized in S1 Table. According to BSDS, 55% (11 out of 20) of PMDD subjects screened positive for bipolar disorder [1]. According to HCL-32, 13 PMDD subjects (65%) demonstrated hypomanic features. In general, this result is consistent with the high prevalence of bipolar disorder in women with PMDD [2]. However, none of PMDD subjects met criteria for bipolar disorder according to MDQ, as none indicated ‘moderate’ or ‘serious’ problems related to the described symptoms [3] .

**S1 Table. Questionnaire scores in women with PMDD.**

| Scales | PMDD group  Mean (S.D.)  [range] |
| --- | --- |
|  |  |
| BDI (follicular)  N_PMDD_ = 20 | 8.35 (7.42)  [2-29] |
| BDI (luteal)  N_PMDD_ = 20 | 18.25 (9.69)  [4-44] |
| BSDS  N_PMDD_ = 20 | 12.35 (5.43)  [2-24] |
| MDQ  N_PMDD_ = 20 | 6.50 (3.58)  [0-11] |
| HCL-32  N_PMDD_ = 20 | 14.8 (6.88)  [1-24] |

N – number of subjects; S.D. – standard deviation; BSDS – Bipolar Spectrum Diagnostic Scale; MDQ – Mood Disorder Questionnaire; HCL-32 – Hypomania Check List; BDI – Beck's Depression Inventory

It has been previously demonstrated that about 23% of women with PMDD suffer from concurrent major depressive disorder [2]. BDI scores in our PMDD sample are consistent with this finding: when assessed during the asymptomatic period (follicular phase), 30% (6 out of 20) of PMDD subjects had mild-to-moderate depression [4]. In contrast, during the premenstrual period (luteal phase), 90% (18 out of 20) of women with PMDD were rated as having mild-to-moderate or more severe depression according to BDI.

**Correlations between the levels of steroid hormones and GR parameters**

**S2 Table. Partial Spearman’s correlations between steroid hormones (estradiol, progesterone) and gamma response (GR) Power and Frequency adjusted for Age in the two groups of participants (control, PMDD)**. Correlations with p<0.05 (uncorrected for multiple comparisons) are highlighted in bold.

| ***A.*** *Estradiol, follicular phase* | | |
| --- | --- | --- |
| Grating’s motion velocity | Control group (N=27) | PMDD group (N=20) |
| *GR power* | | |
| Static, 0 °/s | r=-0.15, p=0.47 | r=-0.10, p=0.70 |
| Slow, 1.2 °/s | r=-0.17, p=0.40 | r=0.00, p=0.99 |
| Medium, 3.6 °/s | r=-0.17, p=0.41 | r=-0.12, p=0.62 |
| Fast, 6.0 °/s | r=-0.13, p=0.53 | r=-0.01, p=0.97 |
| *GR frequency* | | |
| Static, 0 °/s | r=0.29, p=0.16 | r=0.26, p=0.29 |
| Slow, 1.2 °/s | r=0.33, p=0.097 | r=0.20, p=0.41 |
| Medium, 3.6 °/s | **r=0.40, p=0.045** | r=-0.05, p=0.84 |
| Fast, 6.0 °/s | **r=0.41, p=0.037** | r=-0.05, p=0.85 |

| ***B.*** *Estradiol, luteal phase* | | |
| --- | --- | --- |
| Grating’s motion velocity | Control group (N=27) | PMDD group (N=20) |
| *GR power* | | |
| Static, 0 °/s | r=0.20, p=0.33 | r=-0.16, p=0.52 |
| Slow, 1.2 °/s | r=-0.03, p=0.88 | r=-0.09, p=0.71 |
| Medium, 3.6 °/s | r=-0.01, p=0.96 | r=-0.07, p=0.77 |
| Fast, 6.0 °/s | r=0.18, p=0.37 | r=-0.02, p=0.93 |
| *GR frequency* | | |
| Static, 0 °/s | r=0.05, p=0.82 | r=0.35, p=0.14 |
| Slow, 1.2 °/s | r=0.12, p=0.56 | r=0.32, p=0.18 |
| Medium, 3.6 °/s | r=0.05, p=0.81 | r=0.42, p=0.07 |
| Fast, 6.0 °/s | r=0.04, p=0.85 | r=0.35, p=0.15 |

| ***C.*** *Progesterone, luteal phase* | | |
| --- | --- | --- |
| Grating’s motion velocity | Control group (N=27) | PMDD group (N=20) |
| *GR power* | | |
| Static, 0 °/s | r=-0.04, p=0.84 | r=-0.13, p=0.59 |
| Slow, 1.2 °/s | r=-0.25, p=0.21 | r=-0.05, p=0.84 |
| Medium, 3.6 °/s | r=-0.27, p=0.19 | r=-0.05, p=0.83 |
| Fast, 6.0 °/s | r=0.07, p=0.78 | r=-0.01, p=0.96 |
| *GR frequency* | | |
| Static, 0 °/s | r=-0.12, p=0.57 | r=0.19, p=0.43 |
| Slow, 1.2 °/s | r=0.00, p=0.97 | r=0.13, p=0.60 |
| Medium, 3.6 °/s | r=-0.05, p=0.82 | r=-0.03, p=0.91 |
| Fast, 6.0 °/s | r=-0.05, p=0.80 | r=0.05, p=0.84 |

**Gamma Suppression Slope (GSS)**

**GSS: method of calculation**

In the previous studies [5–7], we estimated magnitude of gamma response (GR) suppression as a function of a change in the drift rate of high-contrast visual gratings (1.2°/s, 3.6°/s, 6.0°/s). The *Gamma Suppression Slope* (GSS) - the coefficient of regression of the weighted GR power to velocity - was calculated using the ‘fitlm’ Matlab function: fitlm (x, y, ‘y~x1–1’), where x = [1.2, 3.6, 6.0] corresponds to velocity of motion, y = [0, Power_Medium_/Power_Slow_–1, Power_Fast_/Power_Slow_–1] corresponds to GR power, and ‘y~x1–1’ sets the intercept of the regression line to zero. The resulting regression coefficient b is equal to zero in the case of a constant response power in the three experimental velocity conditions (i.e., ‘no suppression’) and is proportionally more negative in case of stronger velocity-related suppression of the GR.

**High correlation between GSS and GR suppression**

There was a strong correlation between the *GSS* and the *GR suppression* estimated in the present study as a normalized difference between the ‘slow’ and the ‘medium’ velocity condition (N=47; luteal Pearson’s r=-0.90, follicular Pearson’s r=-0.86, p’s<1e-13).

**GSS is not significantly different between the groups but does predict symptom severity in PMDD**

Unlike GR suppression index, the GSS did not differentiate between PMDD and control participants (Student’s t-test; follicular: t(45)=0.79, p=0.44; luteal: t(45)=1.58, p=0.12). However, similarly to GR suppression index*,* GSS correlated with the same-day PMS score in PMDD subjects during the luteal phase (N_PMDD_=18, Pearson’s r=0.51, p=0.03). Again, lower GR suppression, reflected by a less negative GSS, characterized PMDD women with more severe luteal PMS on the day of the investigation.

**Correlation between gamma suppression slope (GSS) and perceptual spatial suppression**
**S3 Table.** **Spearman’s correlations between GSS and spatial suppression index (SSI).**

| MC phase | Control group  (N=26*) | PMDD group  (N=19*) | Difference between correlation coefficients** |
| --- | --- | --- | --- |
| Follicular | **r=-0.45, p=0.02** | r=-0.01, n.s. | p=0.14 |
| Luteal | r=-0.32, p=0.1 | r=0.20, n.s. | p=0.11 |

N – number of subjects; MC – menstrual cycle. Significant correlations and differences are highlighted in bold.

* One PMDD and one control participant were excluded because of the persistent illusion of reversed motion during the presentation of the large grating, which did not allow SSI to be estimated.
** Two-tailed.

**Detailed group comparison on GR power and GR frequency**


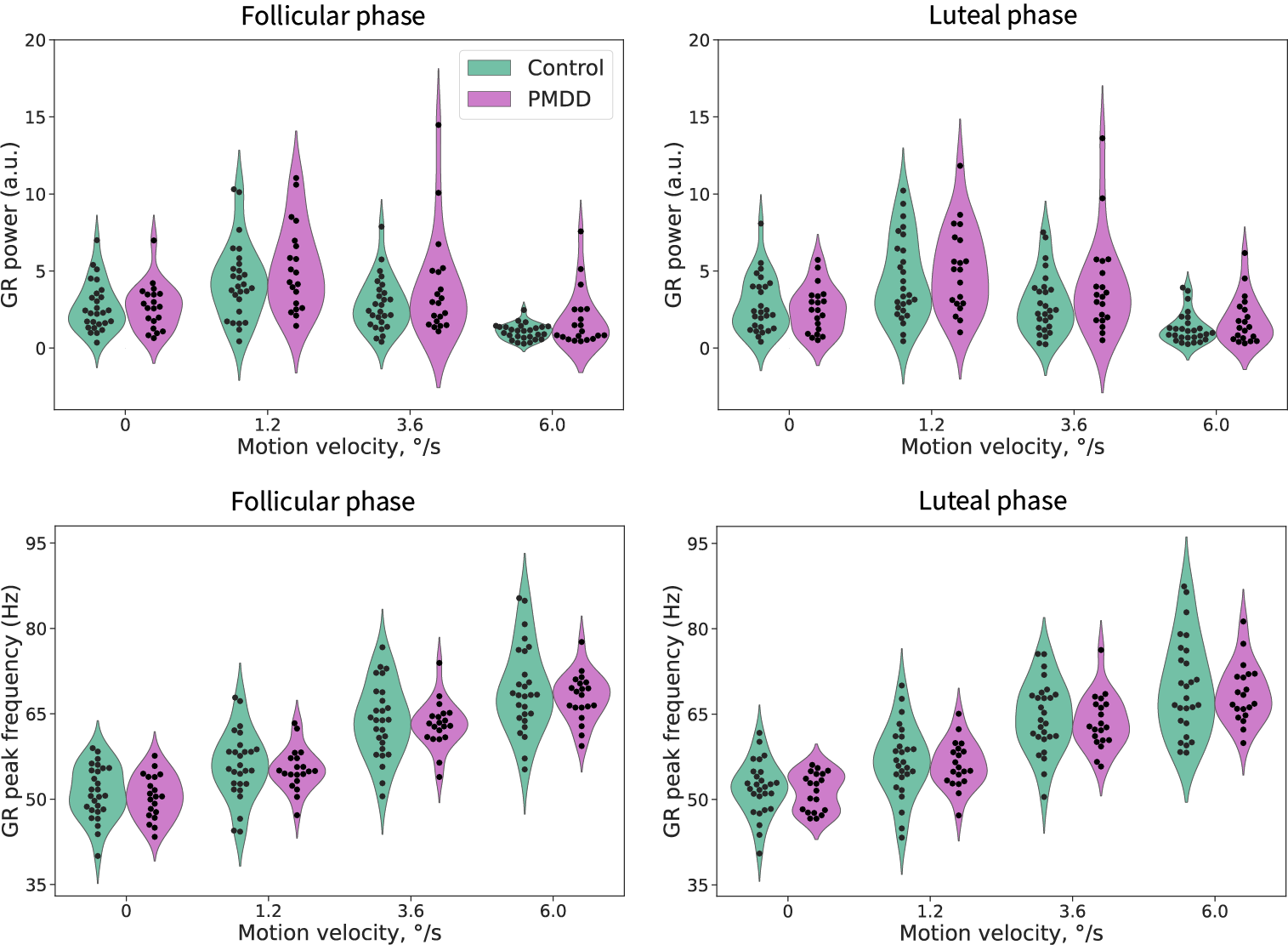
**S1 Fig. Violin plots for gamma response (GR) power (upper panel) and GR frequency (lower panel).** Note that the group differences were not significant for all of these GR parameters (t-test, all p’s>0.12; uncorrected for multiple comparisons).


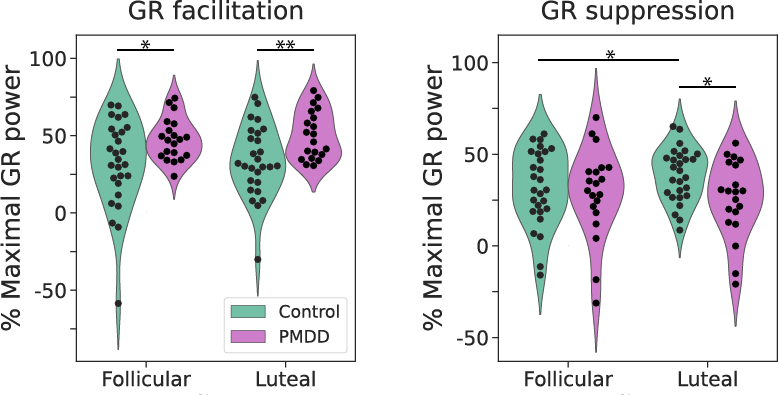


**S2 Fig. Violin plots of gamma response (GR) suppression and GR facilitation scores.**

**References**

1. Ghaemi SN, Miller CJ, Berv DA, Klugman J, Rosenquist KJ, Pies RW. Sensitivity and specificity of a new bipolar spectrum diagnostic scale. J Affect Disord. 2005;84(2–3):273–7.

2. de Carvalho AB, Cardoso T de A, Mondin TC, da Silva RA, Souza LD de M, Magalhães PV da S, et al. Prevalence and factors associated with Premenstrual Dysphoric Disorder: A community sample of young adult women. Psychiatry Res. 2018;268:42–5.

3. Hirschfeld RMA, Williams JBW, Spitzer RL, Calabrese JR, Flynn L, Keck J, et al. Development and validation of a screening instrument for bipolar spectrum disorder: The mood disorder questionnaire. Am J Psychiatry. 2000;157(11):1873–5.

4. Beck AT, Steer RA, Carbin MG. Psychometric properties of the Beck Depression Inventory: Twenty-five years of evaluation. Clin Psychol Rev. 1988;8(1):77–100.

5. Manyukhina VO, Rostovtseva EN, Prokofyev AO, Obukhova TS, Schneiderman JF, Stroganova TA, et al. Visual gamma oscillations predict sensory sensitivity in females as they do in males. Sci Rep. 2021;11(1):1–16.

6. Orekhova EV., Stroganova TA, Schneiderman JF, Lundström S, Riaz B, Sarovic D, et al. Neural gain control measured through cortical gamma oscillations is associated with sensory sensitivity. Hum Brain Mapp. 2019;40(5):1583–93.

7. Orekhova EV., Rostovtseva EN, Manyukhina VO, Prokofiev AO, Obukhova TS, Nikolaeva AY, et al. Spatial suppression in visual motion perception is driven by inhibition: Evidence from MEG gamma oscillations. Neuroimage. 2020;213:116753.
