# Supplementary material for: Altered visual cortex excitability in premenstrual dysphoric disorder: Evidence from magnetoencephalographic gamma oscillations and perceptual suppression
Source: PLoS One. 2022 Dec 30;17(12):e0279868. doi: 10.1371/journal.pone.0279868 (PMC9803314; doi:10.1371/journal.pone.0279868)
Supplement: S2 File — (DOCX) [file pone.0279868.s004.docx]

**Results of bipolar disorder and depression questionnaires in women with PMDD**

The bipolar and depression questionnaire scores for the PMDD group are summarized in S1 Table. According to BSDS, 55% (11 out of 20) of PMDD subjects screened positive for bipolar disorder [1]. According to HCL-32, 13 PMDD subjects (65%) demonstrated hypomanic features. In general, this result is consistent with the high prevalence of bipolar disorder in women with PMDD [2]. However, none of PMDD subjects met criteria for bipolar disorder according to MDQ, as none indicated ‘moderate’ or ‘serious’ problems related to the described symptoms [3] .

**S1 Table. Questionnaire scores in women with PMDD.**

| Scales | PMDD group  Mean (S.D.)  [range] |
| --- | --- |
|  |  |
| BDI (follicular)  N_PMDD_ = 20 | 8.35 (7.42)  [2-29] |
| BDI (luteal)  N_PMDD_ = 20 | 18.25 (9.69)  [4-44] |
| BSDS  N_PMDD_ = 20 | 12.35 (5.43)  [2-24] |
| MDQ  N_PMDD_ = 20 | 6.50 (3.58)  [0-11] |
| HCL-32  N_PMDD_ = 20 | 14.8 (6.88)  [1-24] |

N – number of subjects; S.D. – standard deviation; BSDS – Bipolar Spectrum Diagnostic Scale; MDQ – Mood Disorder Questionnaire; HCL-32 – Hypomania Check List; BDI – Beck's Depression Inventory

It has been previously demonstrated that about 23% of women with PMDD suffer from concurrent major depressive disorder [2]. BDI scores in our PMDD sample are consistent with this finding: when assessed during the asymptomatic period (follicular phase), 30% (6 out of 20) of PMDD subjects had mild-to-moderate depression [4]. In contrast, during the premenstrual period (luteal phase), 90% (18 out of 20) of women with PMDD were rated as having mild-to-moderate or more severe depression according to BDI.

**References**

1. Ghaemi SN, Miller CJ, Berv DA, Klugman J, Rosenquist KJ, Pies RW. Sensitivity and specificity of a new bipolar spectrum diagnostic scale. J Affect Disord. 2005;84(2–3):273–7.

2. de Carvalho AB, Cardoso T de A, Mondin TC, da Silva RA, Souza LD de M, Magalhães PV da S, et al. Prevalence and factors associated with Premenstrual Dysphoric Disorder: A community sample of young adult women. Psychiatry Res. 2018;268:42–5.

3. Hirschfeld RMA, Williams JBW, Spitzer RL, Calabrese JR, Flynn L, Keck J, et al. Development and validation of a screening instrument for bipolar spectrum disorder: The mood disorder questionnaire. Am J Psychiatry. 2000;157(11):1873–5.

4. Beck AT, Steer RA, Carbin MG. Psychometric properties of the Beck Depression Inventory: Twenty-five years of evaluation. Clin Psychol Rev. 1988;8(1):77–100.
