# Supplementary material for: Altered visual cortex excitability in premenstrual dysphoric disorder: Evidence from magnetoencephalographic gamma oscillations and perceptual suppression
Source: PLoS One. 2022 Dec 30;17(12):e0279868. doi: 10.1371/journal.pone.0279868 (PMC9803314; doi:10.1371/journal.pone.0279868)
Supplement: S3 File — (DOCX) [file pone.0279868.s005.docx]

**Gamma Suppression Slope (GSS)**

**GSS: method of calculation**

In the previous studies [1–3], we estimated magnitude of gamma response (GR) suppression as a function of a change in the drift rate of high-contrast visual gratings (1.2°/s, 3.6°/s, 6.0°/s). The *Gamma Suppression Slope* (GSS) - the coefficient of regression of the weighted GR power to velocity - was calculated using the ‘fitlm’ Matlab function: fitlm (x, y, ‘y~x1–1’), where x = [1.2, 3.6, 6.0] corresponds to velocity of motion, y = [0, Power_Medium_/Power_Slow_–1, Power_Fast_/Power_Slow_–1] corresponds to GR power, and ‘y~x1–1’ sets the intercept of the regression line to zero. The resulting regression coefficient b is equal to zero in the case of a constant response power in the three experimental velocity conditions (i.e., ‘no suppression’) and is proportionally more negative in case of stronger velocity-related suppression of the GR.

**High correlation between GSS and GR suppression**

There was a strong correlation between the *GSS* and the *GR suppression* estimated in the present study as a normalized difference between the ‘slow’ and the ‘medium’ velocity condition (N=47; luteal Pearson’s r=-0.90, follicular Pearson’s r=-0.86, p’s<1e-13).

**GSS is not significantly different between the groups but does predict symptom severity in PMDD**

Unlike GR suppression index, the GSS did not differentiate between PMDD and control participants (Student’s t-test; follicular: t(45)=0.79, p=0.44; luteal: t(45)=1.58, p=0.12). However, similarly to GR suppression index*,* GSS correlated with the same-day PMS score in PMDD subjects during the luteal phase (N_PMDD_=18, Pearson’s r=0.51, p=0.03). Again, lower GR suppression, reflected by a less negative GSS, characterized PMDD women with more severe luteal PMS on the day of the investigation.

**Correlation between gamma suppression slope (GSS) and perceptual spatial suppression**
**S3 Table.** **Spearman’s correlations between GSS and spatial suppression index (SSI).**

| MC phase | Control group  (N=26*) | PMDD group  (N=19*) | Difference between correlation coefficients** |
| --- | --- | --- | --- |
| Follicular | **r=-0.45, p=0.02** | r=-0.01, n.s. | p=0.14 |
| Luteal | r=-0.32, p=0.1 | r=0.20, n.s. | p=0.11 |

N – number of subjects; MC – menstrual cycle. Significant correlations and differences are highlighted in bold.

* One PMDD and one control participant were excluded because of the persistent illusion of reversed motion during the presentation of the large grating, which did not allow SSI to be estimated.
** Two-tailed.

**References**

1. Manyukhina VO, Rostovtseva EN, Prokofyev AO, Obukhova TS, Schneiderman JF, Stroganova TA, et al. Visual gamma oscillations predict sensory sensitivity in females as they do in males. Sci Rep. 2021;11(1):1–16.
2. Orekhova EV., Stroganova TA, Schneiderman JF, Lundström S, Riaz B, Sarovic D, et al. Neural gain control measured through cortical gamma oscillations is associated with sensory sensitivity. Hum Brain Mapp. 2019;40(5):1583–93.
3. Orekhova EV., Rostovtseva EN, Manyukhina VO, Prokofiev AO, Obukhova TS, Nikolaeva AY, et al. Spatial suppression in visual motion perception is driven by inhibition: Evidence from MEG gamma oscillations. Neuroimage. 2020;213:116753.
