# Supplementary material for: Altered visual cortex excitability in premenstrual dysphoric disorder: Evidence from magnetoencephalographic gamma oscillations and perceptual suppression
Source: PLoS One. 2022 Dec 30;17(12):e0279868. doi: 10.1371/journal.pone.0279868 (PMC9803314; doi:10.1371/journal.pone.0279868)
Supplement: S1 Table — Correlations with p<0.05 (uncorrected for multiple comparisons) are highlighted in bold. (DOCX) [file pone.0279868.s006.docx]

**S2 Table. Partial Spearman’s correlations between steroid hormones (estradiol, progesterone) and gamma response (GR) Power and Frequency adjusted for Age in the two groups of participants (control, PMDD)**. Correlations with p<0.05 (uncorrected for multiple comparisons) are highlighted in bold.

| ***A.*** *Estradiol, follicular phase* | | |
| --- | --- | --- |
| Grating’s motion velocity | Control group (N=27) | PMDD group (N=20) |
| *GR power* | | |
| Static, 0 °/s | r=-0.15, p=0.47 | r=-0.10, p=0.70 |
| Slow, 1.2 °/s | r=-0.17, p=0.40 | r=0.00, p=0.99 |
| Medium, 3.6 °/s | r=-0.17, p=0.41 | r=-0.12, p=0.62 |
| Fast, 6.0 °/s | r=-0.13, p=0.53 | r=-0.01, p=0.97 |
| *GR frequency* | | |
| Static, 0 °/s | r=0.29, p=0.16 | r=0.26, p=0.29 |
| Slow, 1.2 °/s | r=0.33, p=0.097 | r=0.20, p=0.41 |
| Medium, 3.6 °/s | **r=0.40, p=0.045** | r=-0.05, p=0.84 |
| Fast, 6.0 °/s | **r=0.41, p=0.037** | r=-0.05, p=0.85 |

| ***B.*** *Estradiol, luteal phase* | | |
| --- | --- | --- |
| Grating’s motion velocity | Control group (N=27) | PMDD group (N=20) |
| *GR power* | | |
| Static, 0 °/s | r=0.20, p=0.33 | r=-0.16, p=0.52 |
| Slow, 1.2 °/s | r=-0.03, p=0.88 | r=-0.09, p=0.71 |
| Medium, 3.6 °/s | r=-0.01, p=0.96 | r=-0.07, p=0.77 |
| Fast, 6.0 °/s | r=0.18, p=0.37 | r=-0.02, p=0.93 |
| *GR frequency* | | |
| Static, 0 °/s | r=0.05, p=0.82 | r=0.35, p=0.14 |
| Slow, 1.2 °/s | r=0.12, p=0.56 | r=0.32, p=0.18 |
| Medium, 3.6 °/s | r=0.05, p=0.81 | r=0.42, p=0.07 |
| Fast, 6.0 °/s | r=0.04, p=0.85 | r=0.35, p=0.15 |

| ***C.*** *Progesterone, luteal phase* | | |
| --- | --- | --- |
| Grating’s motion velocity | Control group (N=27) | PMDD group (N=20) |
| *GR power* | | |
| Static, 0 °/s | r=-0.04, p=0.84 | r=-0.13, p=0.59 |
| Slow, 1.2 °/s | r=-0.25, p=0.21 | r=-0.05, p=0.84 |
| Medium, 3.6 °/s | r=-0.27, p=0.19 | r=-0.05, p=0.83 |
| Fast, 6.0 °/s | r=0.07, p=0.78 | r=-0.01, p=0.96 |
| *GR frequency* | | |
| Static, 0 °/s | r=-0.12, p=0.57 | r=0.19, p=0.43 |
| Slow, 1.2 °/s | r=0.00, p=0.97 | r=0.13, p=0.60 |
| Medium, 3.6 °/s | r=-0.05, p=0.82 | r=-0.03, p=0.91 |
| Fast, 6.0 °/s | r=-0.05, p=0.80 | r=0.05, p=0.84 |
