# Supplementary material for: Altered visual cortex excitability in premenstrual dysphoric disorder: Evidence from magnetoencephalographic gamma oscillations and perceptual suppression
Source: PLoS One. 2022 Dec 30;17(12):e0279868. doi: 10.1371/journal.pone.0279868 (PMC9803314; doi:10.1371/journal.pone.0279868)
Supplement: Attachment — Submitted filename: PONErev_responses_rev1.docx [file pone.0279868.s008.docx]

**Responses to Reviewer 1 comments**

We are grateful to the reviewer for the positive evaluation of our work. We have corrected typos and grammar, thank you for pointing them out.

• Line 673: it is stated that perceptual surround suppression was not affected in PMDD, but lines 620-627 in the Results section state and discuss that a significant difference was seen. Please clarify.

Thank you for pointing this out. We clarified the statement (lines 698-701):

‘Contrary to our expectations, GR frequency was not affected in women with PMDD. Their perceptual spatial suppression was not altered in the expected direction (i.e., decreased). On the contrary, in the follicular phase, spatial suppression in women with PMDD tended to be stronger than in control women, but it significantly decreased from the follicular to the luteal phase.’

**Responses to Reviewer 2 comments**

We thank the Reviewer for thoughtful feedback and helpful suggestions for improving our manuscript.

**Major points:**

• Throughout the manuscript, the authors assert that their measure of spatial suppression is explained by inhibition in visual cortex, but this point appears somewhat controversial within the literature, and empirical support is equivocal. In contrast to the work that is cited, a number of studies in both humans and animal models have failed to find a strong link between visual center-surround suppression and inhibitory functioning (Liu et al., 2018; Ozeki et al., 2009; Ozeki et al., 2004; Read et al., 2015; Sato et al., 2016; Schach et al., 2021; Schallmo et al., 2018; Schallmo et al., 2020). Some of these have proposed alternative models, wherein withdrawal of recurrent excitation plays a primary role in the suppression phenomenon, with inhibition playing a secondary or supporting role. To some extent, this seems like an issue about the semantic distinction between ‘inhibition’ (i.e., GABAergic hyperpolarization) and ‘suppression’ (i.e., reduced perceptual functioning). Although the authors’ theory regarding the link between motion suppression and inhibition is interesting, it would be prudent to clarify that this is an area of active research, rather than a settled point in the literature.

We agree that spatial suppression is a complex phenomenon, so additional clarification on this issue is really important. Following the Reviewer’s comment, we rewrote the respective part of the discussion (lines 948-966):

‘Contrary to our original hypothesis, which was based on a presumed link between perceptual spatial suppression and cortical inhibition [75,83], spatial suppression was not reduced in women with PMDD. During the follicular phase, our participants with PMDD tended to have even stronger spatial suppression than control women. This trend is opposite to the effects found in elderly individuals [79,80] and people with neuropsychiatric disorders that are associated with a disturbed balance between excitation and inhibition [81–84,99,167] (but for results similar to ours, see [148]), and is not consistent with an assumed reduction of neural inhibition. One possible reason for this unexpected result is that perceptual spatial suppression is a complex phenomenon, and, as Tadin [75] notes, its association with inhibitory dysfunction is not as straightforward as suggested in some studies cited above. Animal studies of neural surround suppression – a decrease in firing rate of cortical neurons with increasing visual stimulus size – have demonstrated the complexity of this phenomenon, which relies on E/I interactions in the primary visual cortex involving multiple inhibitory cell subtypes, as well as on their top-down regulation by higher-tier cortical areas [77,168–170] The balance of neural mechanisms underlying surround suppression is essential for normal perception, while its disruption may lead to an altered perceptual spatial suppression in clinical populations [170].

Given the critical role of top-down influences in surround suppression [168,171] and perceptual suppression itself [172,173], our results rather indicate preserved or even elevated top-down control in the asymptomatic follicular phase in women with PMDD.’

• The issue of co-morbidity between PMDD and bipolar disorder or depression is important, as visual disruptions, including in center-surround tasks, have been observed in these conditions (Golomb et al., 2009; Miller et al., 2003; Ngo et al., 2011; Norton et al., 2016; Schallmo et al., 2015; Yang et al., 2013). This topic is touched on briefly in the limitations section, but is not fully addressed. How might the authors differentiate between specific effects of PMDD versus effects of co-morbid mood disorders?

Thank you for noting this. Indeed, although none of our participants with PMDD had a formal diagnosis of bipolar disorder and only two of them had depression (in remission), we cannot exclude that some had undiagnosed bipolar disorder or depression. Therefore, our study does not allow us to conclude whether the elevated GR facilitation, which was observed in both luteal and follicular phases, is specific for PMDD. Further studies that include the control groups of patients with these conditions and without past or present PMDD are needed. We added the following clarification (lines 862-869):

‘Because our study is limited to a relatively small sample of women with PMDD, some of whom may have had undiagnosed comorbid neuropsychiatric conditions (e.g., depression), it does not allow us to conclude whether their elevated GR facilitation, which was observed in both luteal and follicular phases, is specific for PMDD. Various neuropsychiatric disorders are thought to be associated with constitutively altered regulation of E/I balance in visual cortex [144,145], which in turn can contribute to visual perceptual abnormalities frequently observed in these disorders [81,146–150]. Further studies that include the control groups of patients with these conditions without past or present PMDD are therefore needed.’

On the other hand, the group difference in GR suppression was specific to the PMDD-symptomatic luteal phase and the reduced GR suppression during this phase correlated with severity of premenstrual symptoms in women with PMDD. Therefore, GR suppression abnormalities are more likely to be specific for PMDD (or severe PMS). The same is true for the spatial suppression, because the change (decrease) in SSI from follicular to the luteal phase was observed only in women with PMDD.

• Regarding the GR suppression metric, I am concerned that expressing this value as a ratio distorts the pattern of results. It appears that the PMDD group has higher GR power in both the slow and medium conditions vs. controls, as supported by the significant group * velocity interaction. This overall difference could induce a spurious difference between these two groups when a ratio metric is used. Instead, a subtraction metric appears warranted. See (Curran-Everett, 2013). This comment applies equally to GR facilitation.

Thank you for pointing this out. We added the following text to the Methods (lines 362-373):

‘To quantify changes in GR power from ‘static’ to ‘slow’ and from ‘slow’ to ‘medium’ conditions (see Results to substantiate the use of these contrasts), we also calculated ‘GR facilitation’ and ‘GR suppression’ as ratios:

*GR suppression =* (1 - GR Power_Medium_/GR Power_Slow_)*100%

*GR facilitation =* (1 - GR Power_Static_/GR Power_Slow_)*100%

A ratio metric can exaggerate between-group difference, especially if groups differ in initial value [101], which in this case corresponds to GR power in the ‘slow’ condition. Besides, a ratio yields reliable results only if the relationship between numerator and denominator is a straight line through the origin for at least one of the two groups being compared [101,102]. These two requirements – no difference in initial value and a zero intercept – were fulfilled for both GR indexes (see S1 Fig for the detailed group comparison of GR power). Therefore, the use of GR suppression and GR facilitation ratio metrics was statistically justified.’

Below we provide a more detailed explanation.

**1.** Regarding subtraction metric, i.e., the absolute difference between the ‘second’ and ‘initial’ measurement.

Curran-Everett and Williams [1] note that ‘***absolute change*** is a metric that can be imperfect: the absolute change may depend, in part, on the initial value y_i_’. This dependency was present in our data. There was a high inter-individual variability of GR power values, which can be largely explained by morphological factors (e.g. size and folding of the primary visual cortex). Predictably, the subtraction metric strongly correlates with GR power. For the GR Power_Slow_, the correlations with subtraction metrics range from 0.57 to 0.84 (all p’s<0.0001), depending on the metric (GR_Slow_ - GR_Static_; GR_Slow_ - GR_Medium_) and the phase (luteal, follicular). Therefore, the subtraction metric seems to be not a good choice for the purpose of the present study.

**2.** Regarding the ratio metric that we used, it seems to be a statistically justified measure according to criteria formulated by Curran-Everett and Williams [1].

*First*, the average GR amplitudes in PMDD group were not significantly different from those in Control group, although they look different in the Fig 6a (all p’s>0.13 according to the T-statistics, see S1 Fig for detailed group comparison of GR power). This decreases the likelihood that the group differences in the ratios are due to the group differences in the initial values [1]. Please, note that Velocity*Group interaction for the GR power was rather due to the significant between-group difference in the *changes* of GR power (its rise from the ‘static’ to the ‘slow’ condition, and its fall from the ‘slow’ to the ‘medium’ one (see Fig 6).

*Second*, Curran-Everett and Williams [1] noted that ‘…the slope of the true relationship between absolute change and initial value differs in the two groups, percent change and symmetrized percent change lead us to the same scientific conclusion as analysis of covariance only if the relationship between absolute change and initial value goes through the origin for at least one of the two groups.’ This was exactly the case for all our relational measures (see Fig A below).

**Fig A.** The linear relationship between GR power in the ‘slow’ condition (condition that is characterized by maximal GR power at the group level) and the ‘subtraction metrics’. Note that in all cases, the regression line goes through the origin in the control group.

*Third*, although we used ratios to visualize the group differences in the velocity-related changes in GR power (Fig 6b,c) and to correlate them with the symptoms (Fig 6d), presence of the group differences in the GR power changes (i.e., GR facilitation, GR suppression) was also supported by results of the ANOVA planned comparisons, which are presented in the Results section.

• According to the text in the Results, the authors calculate their suppression index in a way that is not standard in the field; the standard metric is the log of the difference in threshold values (Tadin, 2003; Foss-Feig, 2013), rather than the ratio. Using the current metric makes it difficult to compare the results of this study to the wider literature. However, the methods state that the standard difference metric was used. Please clarify.

We calculated SSI as:

*SSI* = log10(Threshold_LARGE_/ Threshold_SMALL_), which is equivalent to

log10(Threshold_LARGE_) – log10(Threshold_SMALL_)

Both Foss-Feig et al (page 8245 in [2]) and Tadin et al (Figure 3c legend in [3]) calculated the suppression index in the same way: log10(large stimulus threshold) - log10(small stimulus threshold).

Taking log of the difference may occasionally result in NaN values (if the difference is negative).

• The manuscript is overly-long in general, but frustratingly, appears to leave out important details and justification in places (some examples are provided in the Minor points below). I believe the manuscript would benefit from greater use of supplemental materials. Some tables and analyses (e.g., sections 3.1 – 3.3) are less relevant to the main hypotheses than others and could be briefly summarized in the main text, with details in the supplemental text.

Following the Reviewer's comment, we moved a part of these results to the Supplementary Information.

**Minor points:**

• Line 38 – please clarify that suppression refers to GR power, not perception.
Thank you, we corrected this.

• Lines 106 & 110 – please specify GR power rather than frequency.
Thank you, we changed ‘GR’ for ‘GR power’.

• Please provide an explanation for why controls did not also complete the C-PASS.

To make sure that our control participants did not have severe PMS, we asked them to fill out the PMTS-VAS scale. This questionnaire is not as accurate as the C-PASS. However, it does take much less time. Requiring all healthy participants to fill out the C-PASS questionnaire every day for 2 months would greatly increase dropout and leave us with control women with a certain personality bias (those who are persistent enough to follow the requirements without being interested in the result). On the other hand, women with PMDD were very motivated to find out if they met the PMDD criteria. Therefore, most women with severe premenstrual symptoms who began completing the C-PASS continued to do so for 2 menstrual cycles or longer.

We added the following explanation in Materials and Methods (line 168-172):

‘We expected that using the C-PASS among control participants could greatly increase the dropout. It could also lead to a preponderance of control volunteers with certain personality traits (e.g., those who are persistent enough to follow the requirements without being interested in the C-PASS results). Therefore, to ensure that control participants did not have severe PMS, we used a less time-consuming premenstrual tension syndrome scale (PMTS-VAS, [90]).’

• Which ethics body specifically reviewed the study, and how were participants compensated for their time?

We detailed information regarding the Ethical Committee (lines 208-210):

‘The study has been approved by the Ethical Committee of the Moscow State University of Psychology and Education. Subjects were not paid for their participation.’

Website: https://mgppu.ru/project/459.

• Why were 26 vertices used, and are these in the same position for all participants? Were they required to be contiguous?

Following the approach used by Tan and colleagues [4], to estimate GR parameters in the ‘source space’, we averaged the signal over 26 maximal sources chosen individually for each participant. We used this approach in several our previous studies [5–7]. However, considering the field spread, the exact number of vertices used for analysis is not that important. The similar velocity-related dynamics of GR parameters is observed when one maximal vertex is used. Averaging several vertices increases signal-to-noise ratio.

There was no formal requirement for the vertices to be contiguous, but visual inspection of the data revealed that for all participants the selected vertices were spatially contiguous in the surface mesh of each hemisphere.

We introduced clarification in lines 295-299:

‘Among all the four conditions, we then selected individually for each participant the 26 vertices with maximum response power in gamma range (40-80 Hz), and averaged the spectra over these vertices, separately for each condition. Visual inspection of localization results has shown that for all participants the selected vertices were spatially contiguous in the surface mesh of each hemisphere.’

• Why not include motion discrimination data from the 2.5 degree size condition? No justification is given, and these data might be expected to aid the interpretation of the pattern of results, as the pattern of spatial suppression across stimulus sizes is well established in healthy adults.

Our previous study in healthy participants showed that the duration thresholds for 2.5 degree stimulus significantly improved with practice (from the 1^st^ to the 2^nd^ visit), while thresholds for small and large stimuli remain relatively stable (Supplementary Figure S2 in [5]). Because the thresholds for small and large stimuli were sufficient to assess spatial suppression, to avoid introducing an additional poorly controlled factor, we decided not to include medium-size stimuli in the present analysis.

• Data points from individual participants are shown in Figures 2 and 3, which is appreciated, but this is not the case for later figures.

We plotted the individual data for SSI in Fig 7b. However, it would be difficult to present individual GR power and frequency values in all figures. Therefore, we introduced additional supplementary figures that show individual GR power and GR frequency values (S1 Fig) and GR suppression and GR facilitation indices (S2 Fig) for the two groups.

**Fig 7. Dependence of motion direction distinction on the phase of the menstrual cycle (follicular, luteal phase) in control and PMDD participants**. (a) duration thresholds for the small and large stimuli (b) and spatial suppression index (SSI). Error bars represent 95% confidence intervals.

*p<0.05; **p<10^-8^

**S1 Fig. Violin plots for gamma response (GR) power (upper panel) and GR frequency (lower panel).** Note, that the group differences were not significant for all of these GR parameters (Student’s t-test, all p’s>0.12).

**S2 Fig. Violin plots of gamma response (GR) suppression and GR facilitation scores.**

• It appears the correlations in Table 3 were not corrected for multiple comparisons. This should be done, or at least included in addition to uncorrected p-values, given the large numbers of tests reported (16).
We corrected p-values in Table 3.

• Line 641 – The authors say "In control women, the percent of GR suppression from the ‘slow’ to the ‘medium’ condition (Fig 6c) correlated with the SSI" but the correlation is only significant for the follicular phase, please clarify.
We added clarification in lines 662-666:

‘In control women, the percent of GR suppression from the ‘slow’ to the ‘medium’ condition (Fig 6c) correlated with the SSI during the follicular phase (N_Control_=26; Pearson’s r=0.53, p=0.005). During the luteal phase, the correlation was in the same direction, although not significant (N_Control_=26; Pearson’s r=0.31, p=0.12). The difference between correlation coefficients in the follicular and luteal phases in the control group was not significant.’

• Line 647 – Is there a significant group difference in the GR suppression and SSI correlations themselves?

We added this information in lines 671-673:

‘The group difference in correlation coefficients was significant in the follicular phase (p=0.035, two-sided) but not in the luteal phase (p=0.084, two-sided).’

**References:**

1.	Curran-Everett D, Williams CL. Explorations in statistics: The analysis of change. Adv Physiol Educ. 2015;39(1):49–54.

2.	Foss-Feig JH, Tadin D, Schauder KB, Cascio CJ. A substantial and unexpected enhancement of motion perception in autism. J Neurosci. 2013;33(19):8243–9.

3.	Tadin D, Park WJ, Dieter KC, Melnick MD, Lappin JS, Blake R. Spatial suppression promotes rapid figure-ground segmentation of moving objects. Nat Commun. 2019;10(1):1–12.

4.	Tan HRM, Gross J, Uhlhaas PJ. MEG sensor and source measures of visually induced gamma-band oscillations are highly reliable. Neuroimage. 2016;137:34–44.

5.	Orekhova E V., Rostovtseva EN, Manyukhina VO, Prokofiev AO, Obukhova TS, Nikolaeva AY, et al. Spatial suppression in visual motion perception is driven by inhibition: Evidence from MEG gamma oscillations. Neuroimage. 2020;213:116753.

6.	Orekhova E V., Prokofyev AO, Nikolaeva AY, Schneiderman JF, Stroganova TA. Additive effect of contrast and velocity suggests the role of strong excitatory drive in suppression of visual gamma response. PLoS One. 2020;15(2):1–23.

7.	Manyukhina VO, Rostovtseva EN, Prokofyev AO, Obukhova TS, Schneiderman JF,	Stroganova TA, et al. Visual gamma oscillations predict sensory sensitivity in females as they	do in males. Sci Rep. 2021;11(1):1–16.

**Responses to Reviewer 3 comments**

We thank the Reviewer for the positive evaluation of our work. This is especially gratifying, since this submission is the first one that went through the ‘Editorial’ of a scientific journal and was sent to a peer review. We are also grateful to the Reviewer for helpful suggestions to improve our manuscript.

• My only major comment is to perhaps tone down the Abstract a little bit and explicitly state that the main effect that they were looking for is not present (i.e., it is a null result as far as the main prediction is concerned). This does not take anything away from the work. The reasons provided in the Discussion are thorough and could lead to more refined studies in future. Overall, I endorse the publication of this manuscript.

We have now rewritten the abstract and clearly indicated that two of our predictions (i.e., a change in GR frequency and a decrease in perceptual spatial suppression in women with PMDD during the luteal phase) were not confirmed. However, our third prediction, an abnormal in GR power modulation in women with PMDD during luteal phase, was confirmed.

1) Line 75-77: There is an error in qualifying the concentration of GABAA receptors in visual cortex. Authors state “Visual cortex displays the highest concentration of GABAA and GABAB receptors among other brain areas in humans” but the figure 7 and figure 8 of the cited reference [45] show the highest concentration of GABAA in the auditory cortex.

Thank you for pointing this out. Indeed, GABA/benzodiazepine binding sites in the primary visual cortex are more abundant than in the secondary sensory cortical areas, but not in comparison with other primary cortical areas.

We corrected this (lines 75-76):

‘Primary cortical areas, including visual cortex, display high concentrations of GABAergic receptors [45] and neurons [46] and their GABAergic tone is sensitive to fluctuations in ALLO [44].’

2) Figure 1: Does MEG task panel in the figure depict a single trial? If so, it doesn’t match the description of the task in the methods. In the method (line 225), it is described as “Each new trial started with a fixed 1.2 s prestimulus interval…” but in the figure the trial seems to start with the stimulus followed by a prestimulus period. Also, in the figure it seems as if two stimuli are shown in a trial but it’s not the case according to the methods. An arrow indicating the direction of time can be helpful here.

3) The psychophysical task is not very clear from the figure or from the methods. Little more details on the task, especially the structure of a trial in both methods and figure will be helpful.

Fig 1 shows a schematic representation of experiments rather than detailed structure of individual trials. It would be difficult to present all the details of the experiment in this schematic figure. We now provide more detailed description of the experimental design of the psychophysical experiment in the Methods section.

We corrected the figure and legend to make clear that it is a schematic representation of the experimental procedures and analysis pipelines. The experiments in Fig 1 now start with an inter-stimulus intervals.

**Fig 1. Schematic representation of experimental procedures, analysis of MEG (upper panel) and psychophysical data (lower panel).** For a detailed description of the experimental procedures and analysis pipelines, see the Methods section. The procedures were identical between the two visits. ISI – inter-stimulus interval.

I was unable to find reference [98] online, which is cited for the details of the task. From the figure it seems as if stimuli of different sizes are presented in a trial and response is sought only for the one presented at the end? Is that the case?

We apologize for the wrong reference. The correct one is (lines 1292-1294):

Sysoeva OV, Galuta IA, Davletshina MS, Orekhova EV, Stroganova TA. Abnormal Size-Dependent Modulation of Motion Perception in Children with Autism Spectrum Disorder (ASD). Front Neurosci. 2017;11

In one trial only one stimulus was presented, followed by subject's response. We added more information on individual trials during the psychophysical experiment (lines 324-328):

‘Inter-trial interval was 500 ms. In the beginning of each trial a central dot flickered at the screen (50 ms on, 50 ms off, 250 ms on, 150 ms off) followed by the stimulus presentation. The stimuli were 12°, 2.5°, and 1° vertical high-contrast sinusoidal gratings moving at a constant rate of 4°/s in each trial (Fig 1, lower panel). Direction of motion (left or right) was determined randomly for each trial. The participants were asked to indicate the direction of the visual motion by pressing the corresponding (right or left) button. ’

4) Line 618: There seems to be a mistake in the SSI formula. The ratio of the threshold should have been large/small.
Thank you for pointing that out. We corrected this.

5) Line 645: Three is misspelled as tree.

Thank you. Corrected.

6) Line 818-820: Authors argue that GR facilitation in PMDD is unlikely to depend on MC phase related fluctuations in neurosteroids and the effect of neurosteroids on GABAA mediated inhibition because it enhanced in both the phases. However, this alone is not sufficient to rule out the effect of neurosteroid on GABAA mediated tonic inhibition in case there is a tolerance to ALLO in the luteal phase as the authors hypothesize in section 4.2.1. The evidence for unlikely dependence of facilitation on the effects of neurosteroid on GABAA receptor comes from the control group where the facilitation is maintained despite the absence of the tolerance to ALLO.

Thank you for pointing this out. We added the following text to Results and Discussion.

Results (lines 587-589):

‘The magnitude of GR facilitation did not change between MC phases in either the control (Student’s paired t-test, t(52)=0.16, p=0.87) or PMDD group (Student’s paired t-test, t(38)=0.62, p=0.54).’

Discussion (lines 846-849):

‘Because GR facilitation did not change between the follicular and luteal phases in either control or PMDD women, but was elevated in PMDD women during both of these MC phases, it is unlikely to depend on neurosteroid changes in MC and may reflect constitutively elevated excitability of the principal neurons in women with PMDD.’

7) Table 3: Correlations were significant for control women but not for PMDD subjects. Any reason why?

There was no significant difference between the correlation coefficients in the PMDD and control groups for any of these 16 GR parameters (lines 529-530). More subjects are needed to find out if the observed age trends are different in the two groups.

8) Line 256: Is it ‘Humming’ or Hamming window?

Thank you. Corrected.
